# Morphological and Mechanical Property Differences in Trapeziometacarpal Ligaments of Healthy and Osteoarthritic Female Joints

**DOI:** 10.1007/s10439-024-03660-4

**Published:** 2024-12-07

**Authors:** Lizzie Walker, Daniel Gordon, Alexander Chiaramonti, Shangping Wang, Zhaoxu Meng, Dane Daley, Elizabeth Slate, Hai Yao, Vincent D. Pellegrini, Yongren Wu

**Affiliations:** 1 Department of Bioengineering, Clemson University, 68 President Street, BEB 203, Charleston, SC 29425 USA; 2 Department of Orthopaedic Surgery and Rehabilitation, Wake Forest University School of Medicine, Winston Salem, NC USA; 3 Department of Orthopaedics and Physical Medicine, Medical University of South Carolina, Charleston, SC USA; 4 Department of Mechanical Engineering, Clemson University, Clemson, SC USA; 5 Department of Statistics, Florida State University, Tallahassee, FL USA; 6 Department of Orthopaedics, Dartmouth-Hitchcock Medical Center, Lebanon, NH USA

**Keywords:** Trapeziometacarpal osteoarthritis, Carpometacarpal osteoarthritis, Anterior oblique ligament, Trapeziometacarpal ligaments, Mechanical properties, Static stabilizer

## Abstract

**Purpose:**

To identify changes in morphological and mechanical properties in the volar ligament complex (VLC), dorsoradial ligaments (DRL), and posterior oblique ligaments (POL) in healthy and osteoarthritic female trapeziometacarpal (TMC) joints.

**Methods:**

Twenty-four fresh-frozen female cadaveric TMCs were separated into (1) younger healthy/early-stage osteoarthritic, (2) elder healthy/early-stage osteoarthritic, and (3) advanced-stage osteoarthritic groups based on age and Eaton-Littler grading. Stress relaxation and load-to-failure testing were performed to characterize mechanical tensile properties. Light imaging and scanning electron microscopy (SEM)/energy dispersive spectroscopy (EDS) were performed to further assess enthesis structural integrity.

**Results:**

The VLC in advanced-stage osteoarthritic TMCs had attenuated mechanical properties in stress relaxation experiments compared to the elder healthy/early-stage osteoarthritic specimens: Young’s modulus at 20% strain (*P* = 0.044), instantaneous (*P* = 0.023), relaxed (*P* = 0.017) moduli. VLCs in advanced-stage osteoarthritic TMCs also had significantly lower properties in the load-to-failure experiments compared to the younger healthy/early-stage osteoarthritic specimens: stiffness (*P* = 0.048), ultimate load (*P* = 0.017), toughness (*P* = 0.003). Light and SEM/EDS imaging revealed partial detachment and loss of enthesis mineral gradient at VLC metacarpal insertion in advanced-stage osteoarthritic specimens. There were no mechanical or structural changes in the DRL and POL between experiment groups.

**Conclusion:**

VLC morphological and mechanical properties deteriorate across progressively severe osteoarthritis classifications while the DRL and POL remain unchanged. The attenuated mechanical properties of VLCs in advanced-stage osteoarthritic TMCs can be explained by ligament degradation as evidenced by partial detachment and loss of mineral gradient at the metacarpal insertion.

**Supplementary Information:**

The online version contains supplementary material available at 10.1007/s10439-024-03660-4.

## Introduction

**Fig. 1:**
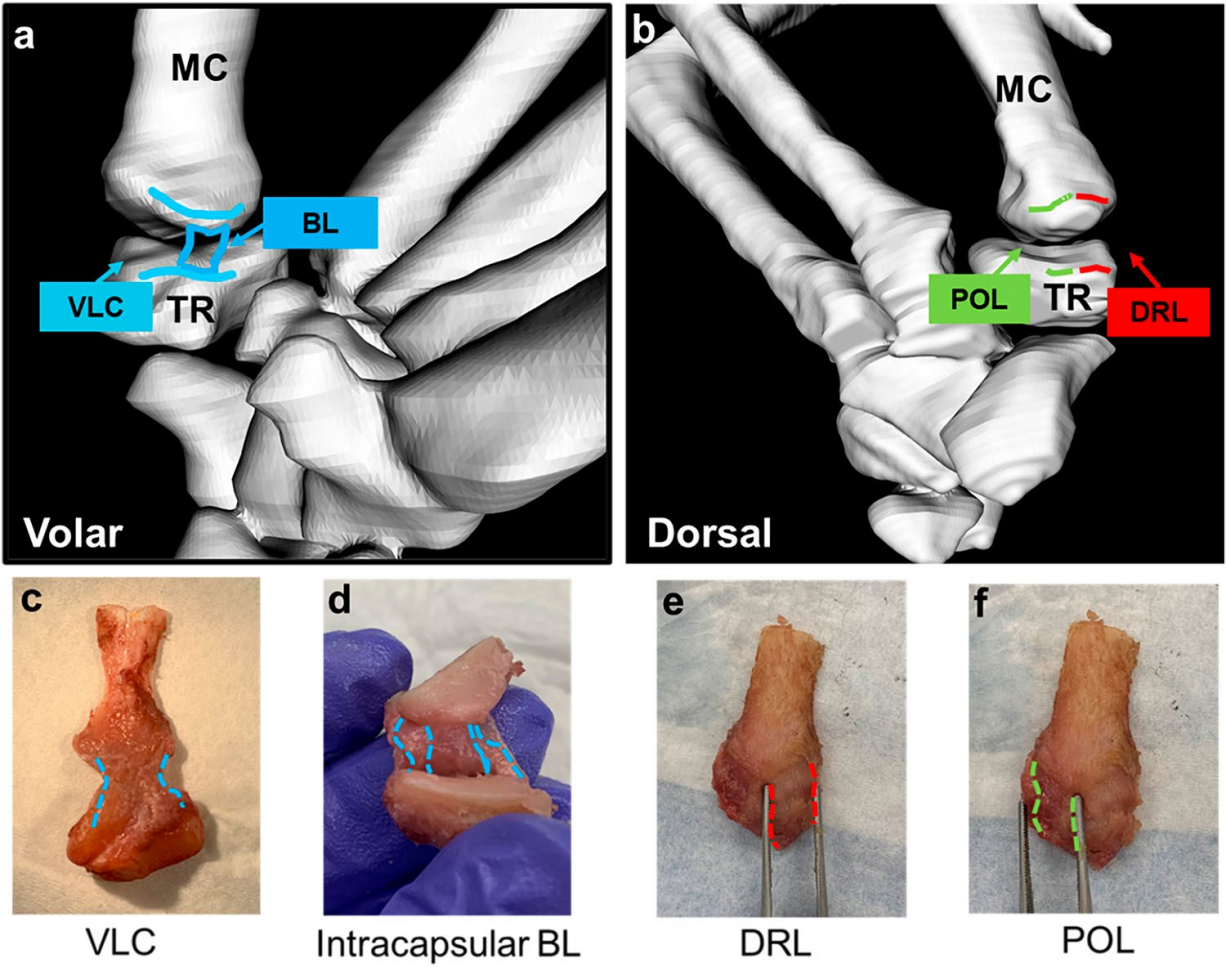
Anatomy of TMC joint: **a** schematic of the volar aspect of TMC joint with ligament insertions and origins, **b** schematic of the dorsal aspect of TMC joint with ligament insertions and origins, **c** dissection of volar aspect of TMC joint, **d** intraarticular point of view of volar aspect to see the beak ligament (BL), and **e, f** dissection of dorsal aspect of TMC joint

The trapeziometacarpal (TMC) joint, also known as the basal joint of the thumb, is one of the most common sites for osteoarthritis (OA) in the upper limb, particularly prevalent in postmenopausal women. Women outnumber men by 10-15 to 1 for basal joint reconstructive procedures due to OA [1–3]. Serving as the foundation of the thumb, the TMC joint is characterized by its wide range of motion and opposability, crucial for daily functional hand tasks such as pinch, grasp, and twist. However, lacking bony stability, it relies on muscles and ligaments for both active and static stabilization, respectively [4]. The principal ligamentous stabilizers for the TMC joint can be separated into volar and dorsal ligaments. Volar ligaments constitute the volar ligamentous complex (VLC) which is comprised of a thin, superficial capsular structure [superficial anterior oblique ligament (sAOL)] and deep, intracapsular beak ligament (BL) [also subsequently described as the deep AOL (dAOL)] that inserts onto the ulnar aspect of the volar beak of the thumb metacarpal, contiguous with the articular surface. Dorsal ligaments include the dorsoradial ligament (DRL), dorsal central ligament (DCL), and posterior oblique ligament (POL) [5–8] (Fig. 1). The DRL and DCL emanate from the dorsal tubercle of the trapezium through the radial and central aspects, respectively, and then insert onto the dorsoradial and central dorsal sides of the first metacarpal [5, 6, 9]. As the ligamentous boundary between the DRL and DCL may not always be clearly visualized, previous studies often combined DCL together with DRL as one ligamentous complex [7, 10]. The POL originates from the ulnar side of the dorsal tubercle of the trapezium and broadly inserts onto the ulnar side of the first metacarpal [6].

Previous research found that, in comparison to the VLC, the dorsal ligaments, particularly the DRL, are generally stiffer and tougher with higher ultimate load [10, 11]. Other anatomical research revealed that the dorsal ligaments, including DRL and POL, were uniformly stout and robust, with thicker ligaments morphometrically and higher cellularity histologically than the VLC [6, 7]. Additionally, they were innervated with more mechanoreceptors, and there was a more evident alteration in mechanoreceptor population and distribution that accompanies OA development [12, 13]. These studies suggested that the dorsal ligaments, identified as key joint stabilizers, may play a critical role in the progression of TMC OA [6, 10, 11]. These quantitative studies, however, do not provide a clear tie explaining any ligament’s role in TMC OA progression, other than the changes in mechanoreceptor volume observed during OA development. By contrast, previous histopathology studies documented evidence of BL fibrocartilage enthesis detachment, particularly at its intracapsular metacarpal insertion in osteoarthritic TMC joints that is otherwise not evident in healthy TMC joints. Additionally, these studies have observed concurrent adjacent articular cartilage degeneration around the BL detachment site, predominantly in female osteoarthritic TMC specimens [8, 14]. Pathomechanical studies further demonstrated that, in the healthy/early-stage osteoarthritic TMCs, articular surface contact and eburnation are more common on the volar side of the metacarpal adjacent to the BL insertion. As the OA progressed, these patterns spread dorsoradially, suggesting a potential etiological relationship between deterioration of the VLC, specifically the BL, and TMC OA progression [8, 15, 16].

To the best of our knowledge, the changes in ligamentous tensile biomechanical properties during the progression of TMC OA, as well as the alterations in structural integrity and mineral gradient, particularly at the insertion entheses that bridges the ligaments and metacarpal/trapezium bones, are still unknown for the principal ligamentous stabilizers, including both VLC (sAOL and BL) and dorsal (DRL and POL) ligaments. Therefore, the objectives of this study are to (1) quantify the tensile mechanical properties of major TMC ligaments including the volar ligamentous complex (VLC; sAOL and BL), dorsoradial ligament (DRL), and posterior oblique ligament (POL) from healthy and osteoarthritic joints, and (2) illustrate tissue structure and mineral compositions at the insertion entheses of each ligament. We hypothesize that the VLC exhibits attenuated mechanical properties in the osteoarthritic joints, attributed to BL enthesis degeneration as evidenced by structural and compositional changes, particularly at its intracapsular metacarpal insertion. Meanwhile, the DRL and POL are postulated to maintain similar magnitudes of tensile properties, with intact enthesis structure and mineral composition in both healthy and osteoarthritic joints. Given the scarcity of human cadaveric specimens, we chose to concentrate on the female population as it is the demographic most affected [1–3]. This approach aimed to minimize human subject variation and ensure a high-quality baseline comparison of the volar and dorsal ligaments in females under both healthy and osteoarthritic stages. The goal of this project is to further understand the pathomechanics of TMC OA with a focus on the surrounding ligaments, specifically in the female population.

## Materials and Methods

### Specimen Preparation

Twenty-four fresh frozen cadaver hands were obtained through an organ procurement organization (approved by Institutional Review Board for Human Research at Medical University of South Carolina). The specimens were all female, consisted of both right and left hands, and ranged in age from 28–73. Specimens were segmented just proximal or just distal to the elbow joint and stored in a − 20 °C freezer until dissection. Specimens were left at room temperature for 12 hours to thaw prior to dissections. Lateral and Robert’s view radiographs were obtained for each specimen and blindly graded by our clinical collaborators (D.D. and A.C.) following the Eaton–Littler classification system [17]. The hand specimens were organized into three experiment groups: (1) younger specimens with early-stage or no OA (n = 8, ≤ 45 years, Eaton–Littler Grade 0–II), (2) elder specimens with early-stage or no OA (n = 8, > 45 years, Eaton–Littler Grade 0–II), and (3) any specimens with advanced-stage osteoarthritic changes (n = 8, no age requirement, Grade III–IV) (Table 1, Online Resource 1). Six specimens per group were randomly selected for mechanical testing, and the remaining two specimens per group were used for scanning electron microscopy.

To gain access to the TMC joint, the adjacent scaphoid, trapezoid, index metacarpal, and thumb proximal phalanx were carefully dissected out using a surgical scalpel. Excess soft tissue (muscle, fascia, fat) covering the joint capsule was then cleared away on the volar side and dorsal side of the joint until the joint capsule of the TMC was accessible. The VLC, DRL, and POL were identified under a dissection microscope by our clinical collaborators (A.C., D.D., V.P.) based on the anatomical features defined in literature [6–8]. Next, bone-ligament-bone complexes of each ligament of interest with the corresponding proximal and distal bony insertion sites were created using an oscillating saw with a small segment blade to first make a coronal cut through the joint, along the plane of the abductor pollicis longus, separating the volar and dorsal sides of the joint before using a micro-saw to dissect out individual ligaments (Fig. 1c–e) [6, 10, 11, 18]. The VLC was not dissected into separate sAOL and BL components, as some osteoarthritic specimens demonstrated degeneration of BLs, making it difficult to isolate the individual ligaments. The length of each ligament was measured from the MC insertion to the TR insertion while the complex was held in passive tension. Width and thickness of each ligament were then measured at the midplane of each ligament. All measurements were performed with a digital micro caliper (0.001 mm precision). To assess the tissue structural integrity (presence or lack of ligament detachment), all ligaments were imaged under a trinocular stereo zoom microscope (AmScope) at 0.8×, 1.25×, and 2× magnification at both the metacarpal and trapezium entheses, as well as the mid-plane of the ligament.

### Mechanical Testing

**Fig. 2:**
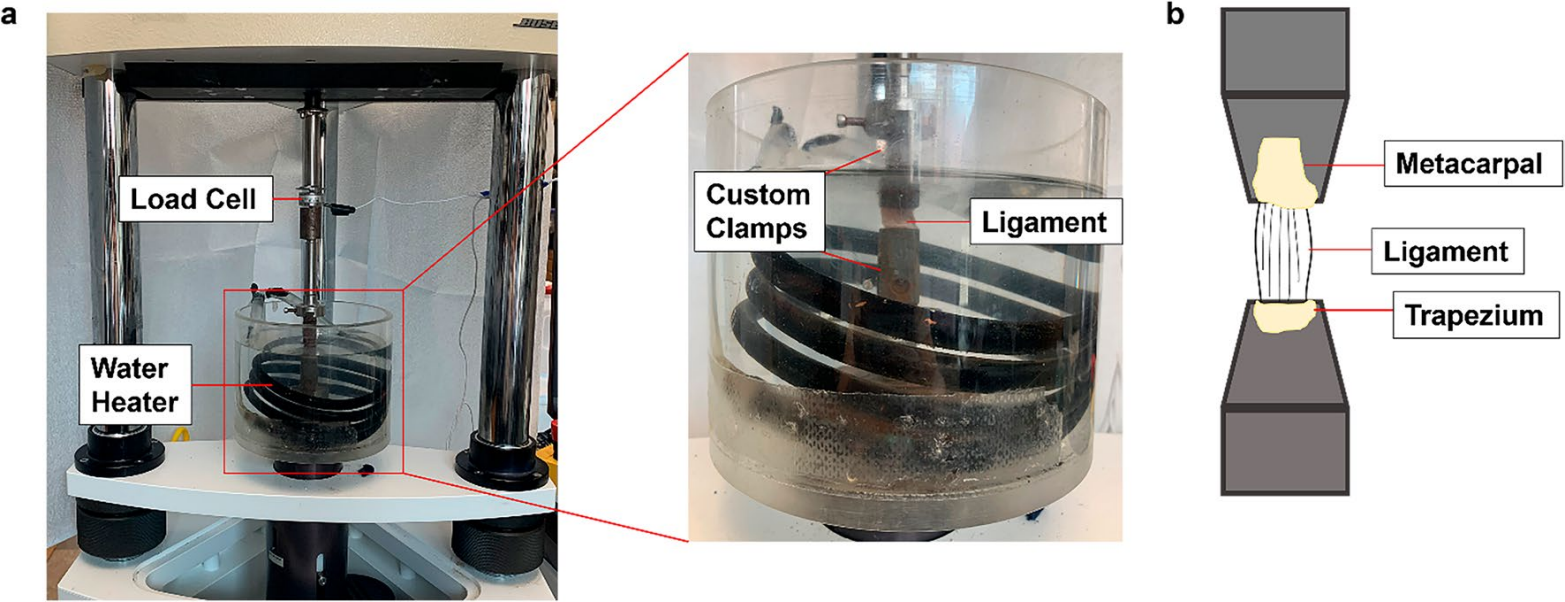
Mechanical testing setup: (**a**) stress relaxation setup with temperature-controlled PBS bath, (**b**) schematic of ligament loaded in clamp

**Fig. 3:**
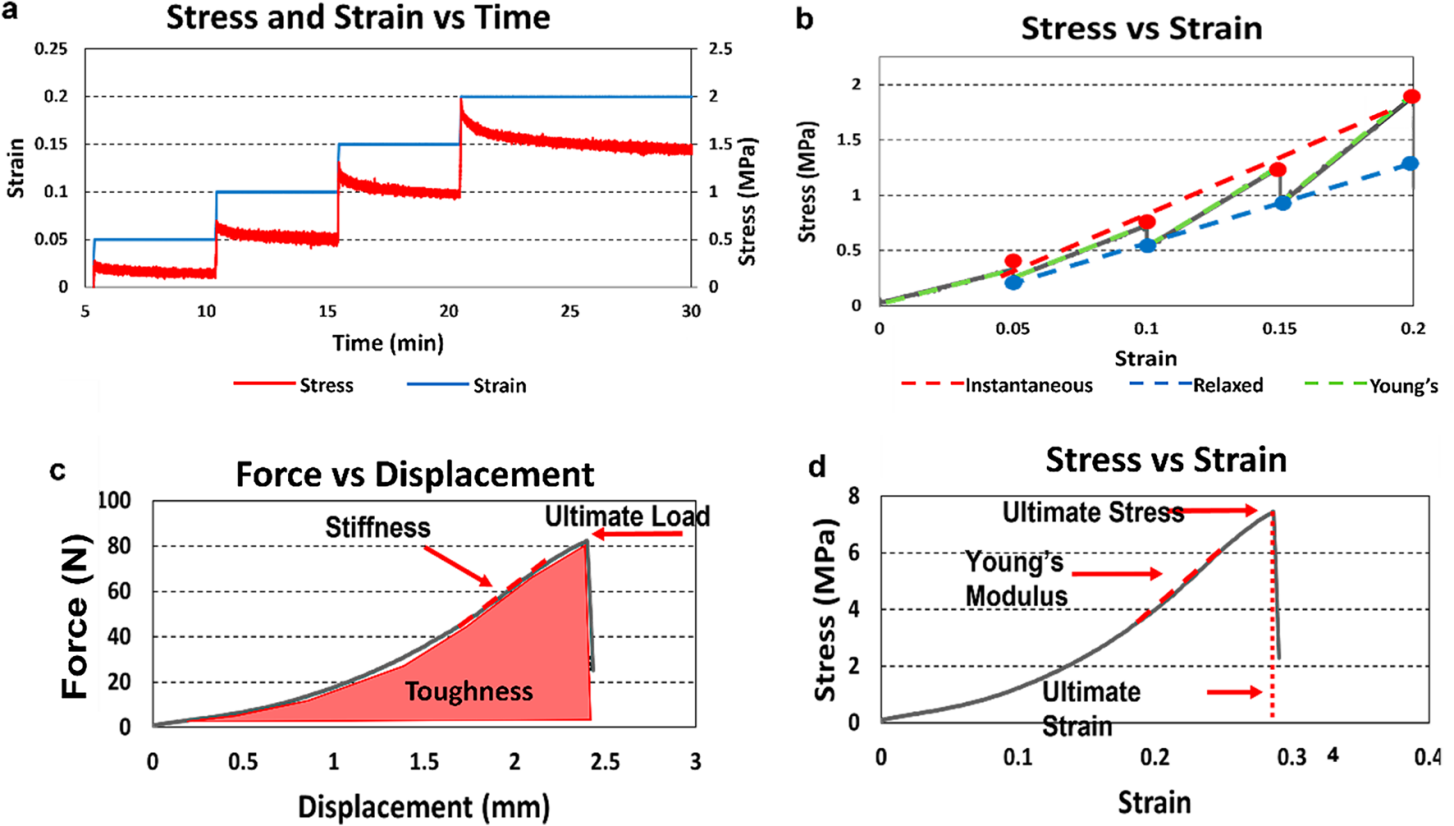
Mechanical testing protocol: (**a**) stress-relaxation tests performed at strain increments of 5%, 10%, 15%, and 20% strain, (**b**) stress vs strain plot where instantaneous, relaxed, and Young’s moduli were calculated, (**c**) load-to-failure force vs displacement plot where stiffness, ultimate load, and toughness were calculated, and (**d)** load-to-failure stress vs strain plot where Young’s modulus, ultimate stress, and ultimate strain were calculated

Ligament complexes were gripped by custom machined clamps, utilizing cerclage wire and cotton fabric to prevent slipping. The cerclage wire was affixed to the metacarpal and trapezium in a figure-eight construct and the cotton fabric was wrapped around the wired ends for the custom clamps to grip. This was found to be the most effective way to prevent slippage and premature bone rupture [11]. Specimens were carefully gripped on the metacarpal and trapezium bones, ensuring that the grip-to-grip alignment corresponded to the ligamentous fiber orientation. Stress-relaxation tests were performed using a Bose tensile testing machine (ElectroForce 3220; TA Instruments, New Castle, DE) in 37 °C PBS bath with a custom-made chamber and clamp setup (Fig. 2). Specimens were initially loaded to 0.5 N prior to any preconditioning or mechanical testing. The ligaments were preconditioned at 4% strain for 10 cycles at 0.5 Hz [10, 11, 19, 20]. Incremental stress-relaxation tests were performed at 5%, 10%, 15%, and 20% strain applied with a ramp-strain rate of 2% strain per second [19, 20]. Strain was calculated by the change in grip-to-grip distance divided by initial specimen grip-to-grip length. There was a relaxation period of 300 seconds after the 5%, 10%, and 15% strain, and 600 seconds after 20% strain, to ensure the ligament reached equilibrium before moving to the next ramp-load phase. An asymptotic approach was used to determine when equilibrium was reached where the change in stress over time is negligible at equilibrium. Instantaneous modulus, relaxed modulus, and Young’s modulus for each ramp-loading phase were obtained from stress–strain curves. Instantaneous and relaxed moduli were calculated as the slope of the best-fit line through the local maxima and minima of the stress–strain curves, respectively. Young’s modulus was calculated as the slope of the best-fit line during the ramp-loading phase [19–21] (Fig. 3a, b).

Next, load-to-failure testing was performed at a ramp-strain rate of 10% strain per second using the Bose tensile testing machine (Fig. 2b). Stiffness, ultimate load, and toughness were calculated based on the load-displacement curves (Fig. 3c). Young’s modulus, ultimate tensile stress, and ultimate strain were calculated based on the stress-strain curves (Fig. 3d). To capture the ligament failure pattern, a paint splatter technique [22] was used to mark ligaments with a pattern that was optically trackable by a DSLR camera (EOS Rebel T6; Canon). Local strain analysis was performed using a digital image correlation (DIC) program (version 1.2; Ncorr, Georgia Tech University, Atlanta, GA). Within the program, 16 seeds with a radius of 10 pixels were placed in a 4x4 grid pattern to focus on calculating displacements and strains occurring at metacarpal and trapezium insertion sites as well as the midplane of each ligament. The program produced contour plots for displacement in the u and v direction, strain in the x and y direction, as well as shear strain.

### Tissue Imaging

Mineral composition distributions along each enthesis and midplane were further determined using a scanning electron microscopy (SEM) (S-3700N; Hitachi) coupled with energy dispersive spectroscopy (EDS) (AZtec; Oxford Instruments). Prior to SEM/EDS imaging, the TMC bone-ligament-bone complex was separated into three compartments including two entheses (i.e., metacarpal and trapezium) and a midplane using a surgical scalpel. Each compartment was further trimmed using a freezing stage sliding microtome (SM2400, Lecia Biosystems) to obtain a flat surface to ensure best image quality. The prepared specimens were then placed in a desiccator for two to three days until fully dehydrated. After sputter-coating with gold at 50 mA for 30-45 s, the specimens were imaged in both secondary and backscatter modes at 10 kV at a magnification of 75x and 500x. EDS analysis, specifically linescan analysis, was used to collect spectra at the metacarpal and trapezium entheses as well as the midplane to characterize calcium and phosphate elemental distributions.

### Statistical Analysis

The effects of OA group (younger healthy/early-stage OA; elder healthy/early-stage OA; advanced-stage OA) and ligament type (VLC, DRL, POL) on ligament dimensions, stress-relaxation, and load-to-failure outcomes were evaluated. Linear mixed-effects models were fit to each outcome, with OA group and ligament type indicators modeled as fixed effects, and donor information included as a random effect to account for within-donor correlation. Effect of hand side (left or right) was initially modeled, but no hand side dependence was found; therefore, it was removed. Where significant differences were seen, pairwise comparisons were made, incorporating a Bonferroni p-value adjustment to account for multiplicity error. All statistical inferences are derived from the fitted mixed-effects model. Statistical differences are reported as *P < 0.05.* Statistical analyses were performed in SPSS (version 28.0.1.0; IBM). Complete statistical details can be found in the Online Resource 1.

## Results

### Morphological Dimensions

**Fig. 4:**
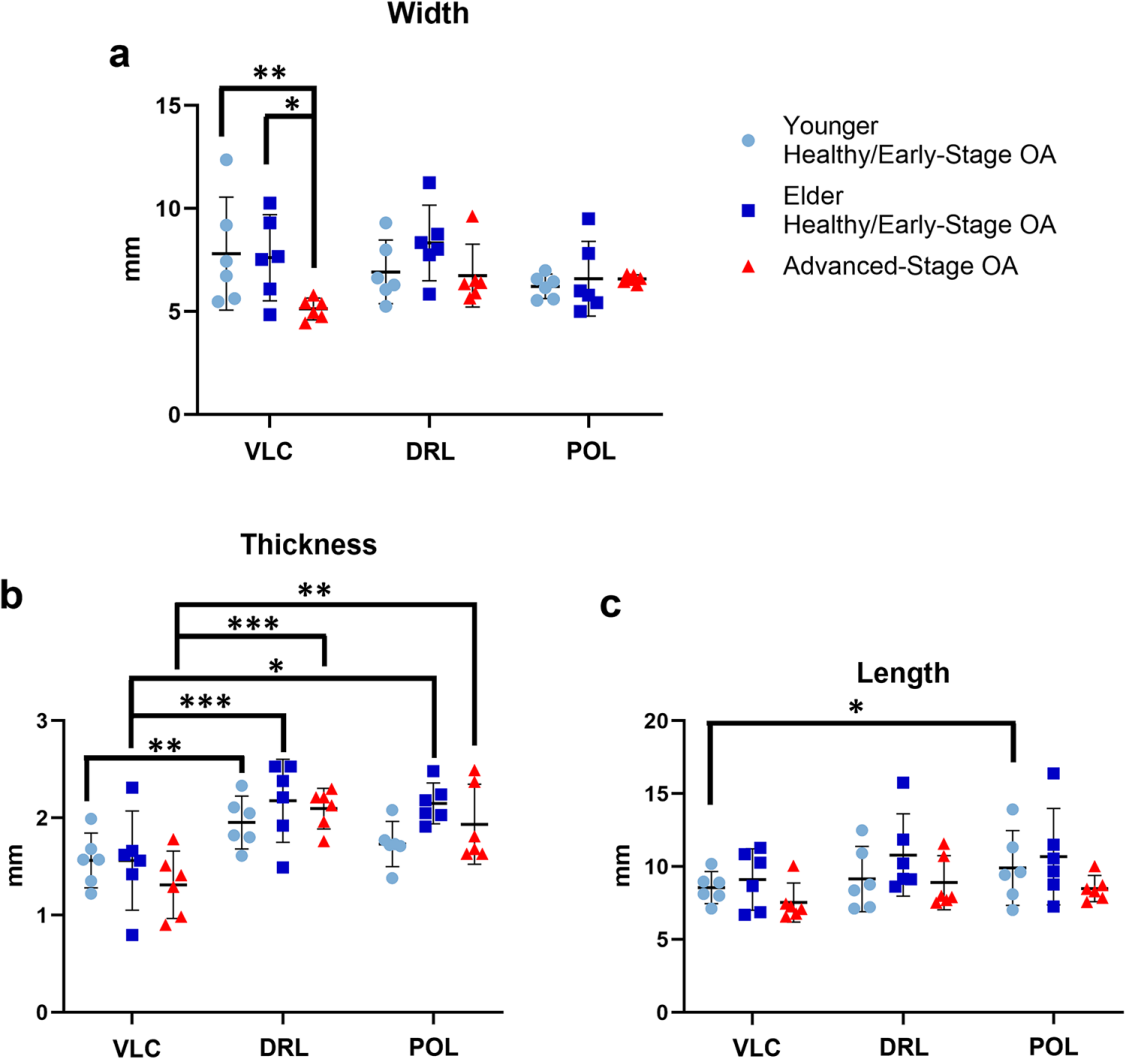
Ligament dimensions: (**a**) width, (**b**) thickness, (**c**) length. Data shown are mean ± 95% confidence interval. *P < 0.05, **P < 0.01, ***P < 0.001

Among disease groups, a decrease of VLC width was found in the advanced-stage OA group (5.25 ± 0.52 mm) when compared to the younger healthy/early-stage OA (7.5 ± 2.28 mm, *P* = 0.009) and elder healthy/early-stage OA group (7.06 ± 1.69 mm, *P* = 0.034) (Fig. 4), while no difference was found in either VLC length or thickness. No difference was found in any dimensions of DRL and POL between OA groups. Among ligament types, VLC had a smaller minimum thickness than that of DRL in all OA groups, including younger healthy/early-stage OA (VLC: 1.44 ± 0.37 mm, DRL: 2.16 ± 0.51 mm, *P* = 0.002), elder healthy/early-stage OA (VLC: 1.38 ± 0.52 mm, DRL: 2.37 ± 0.51 mm, *P* < 0.001), and advanced-stage OA (VLC: 1.20 ± 0.36 mm, DRL: 2.05 ± 0.30 mm, *P* < 0.001) (Fig. 4). Additionally, VLC had a smaller minimum thickness than that of POL in the elder healthy/early-stage OA (VLC: 1.38 ± 0.52 mm, POL: 1.87 ± 0.49 mm, *P* = 0.04) and advanced-stage OA (VLC: 1.20 ± 0.36 mm, POL: 1.94 ± 0.39 mm, *P* = 0.004) (Fig. 4). VLC had a shorter length in the younger healthy/early-stage OA group than that of POL (VLC: 8.38 ± 0.95 mm, POL: 9.91 ± 2.20 mm, *P* = 0.041). All sample means and standard deviations were listed in Table 2 in Online Resource 1. Detailed model-based comparisons of ligament dimensions were listed in Tables 5 and 6 in Online Resource 1.

### Tensile Mechanical Properties

**Fig. 5:**
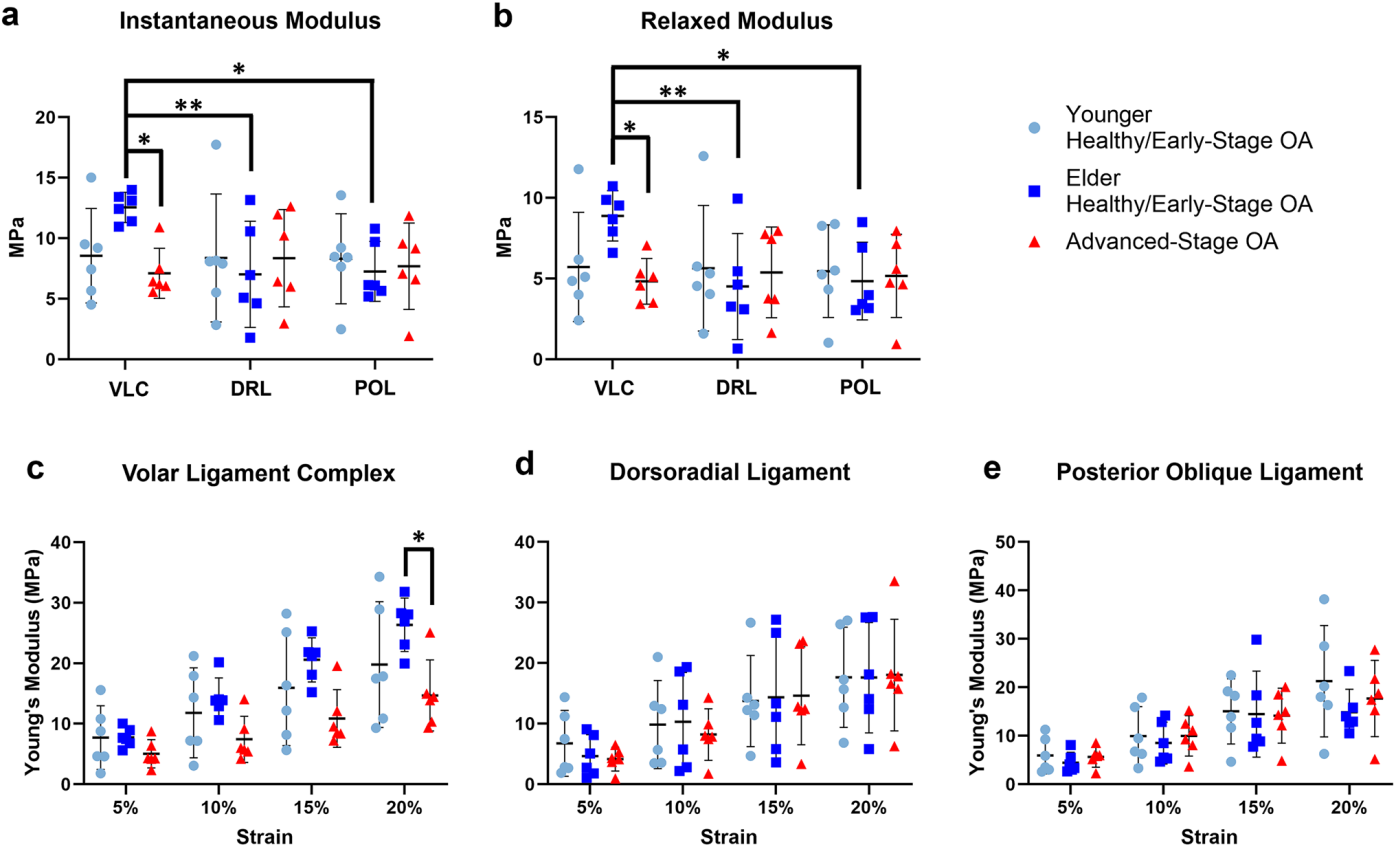
Stress-relaxation results: (**a**) instantaneous moduli, (**b**) relaxed moduli, (**c**) Young’s modulus in VLC, (**d**) Young’s modulus in DRL, and (**e**) Young’s modulus in POL. Data shown are mean ± 95% confidence interval. *P < 0.05, **P < 0.01

Among disease groups, stress relaxation outcomes showed a decrease of VLC Young’s modulus at 20% strain (26.36 ± 4.20 MPa to 14.68 ± 5.59 MPa, *P* = 0.044). A weak decreasing trend in Young’s modulus was also seen at 10% (14.25 ± 3.17 MPa to 7.44 ± 3.63 MPa, *P* = 0.088) and 15% (20.57 ± 3.47 MPa to 10.89 ± 4.55 MPa, *P* = 0.104) strains, as well. A decrease in VLC instantaneous (12.55 ± 1.19 MPa to 7.11 ± 1.97 MPa, *P* = 0.023) and relaxed (8.89 ± 1.48 MPa to 4.83 ± 1.34 MPa, *P* = 0.017) modulus in advanced-stage OA group was also seen when compared to elder health/early-stage OA group (Fig. 5). No significant difference was found in DRL and POL between OA groups. Among ligament types, VLC instantaneous (12.55 ± 1.19 MPa) and relaxed moduli (8.89 ± 1.48 MPa) were higher than the DRL (instantaneous: 7.03 ± 4.17 MPa, *P* = 0.006; relaxed: 4.51 ± 3.13 MPa, *P* = 0.003) and POL (instantaneous: 7.86 ± 2.67 MPa, *P* = 0.05; relaxed: 5.02 ± 2.15 MPa, *P* = 0.025) in the elder healthy/early-stage OA group., Additionally, a weak trend showed that VLC had a higher Young’s modulus at 20% strain (26.36 ± 4.20 MPa) than that of DRL (17.60 ± 8.69 MPa, *P* = 0.089) and POL (16.16 ± 5.33 MPa, *P* = 0.089) in the elder healthy/early-stage OA group. (Fig. 5).

**Fig. 6:**
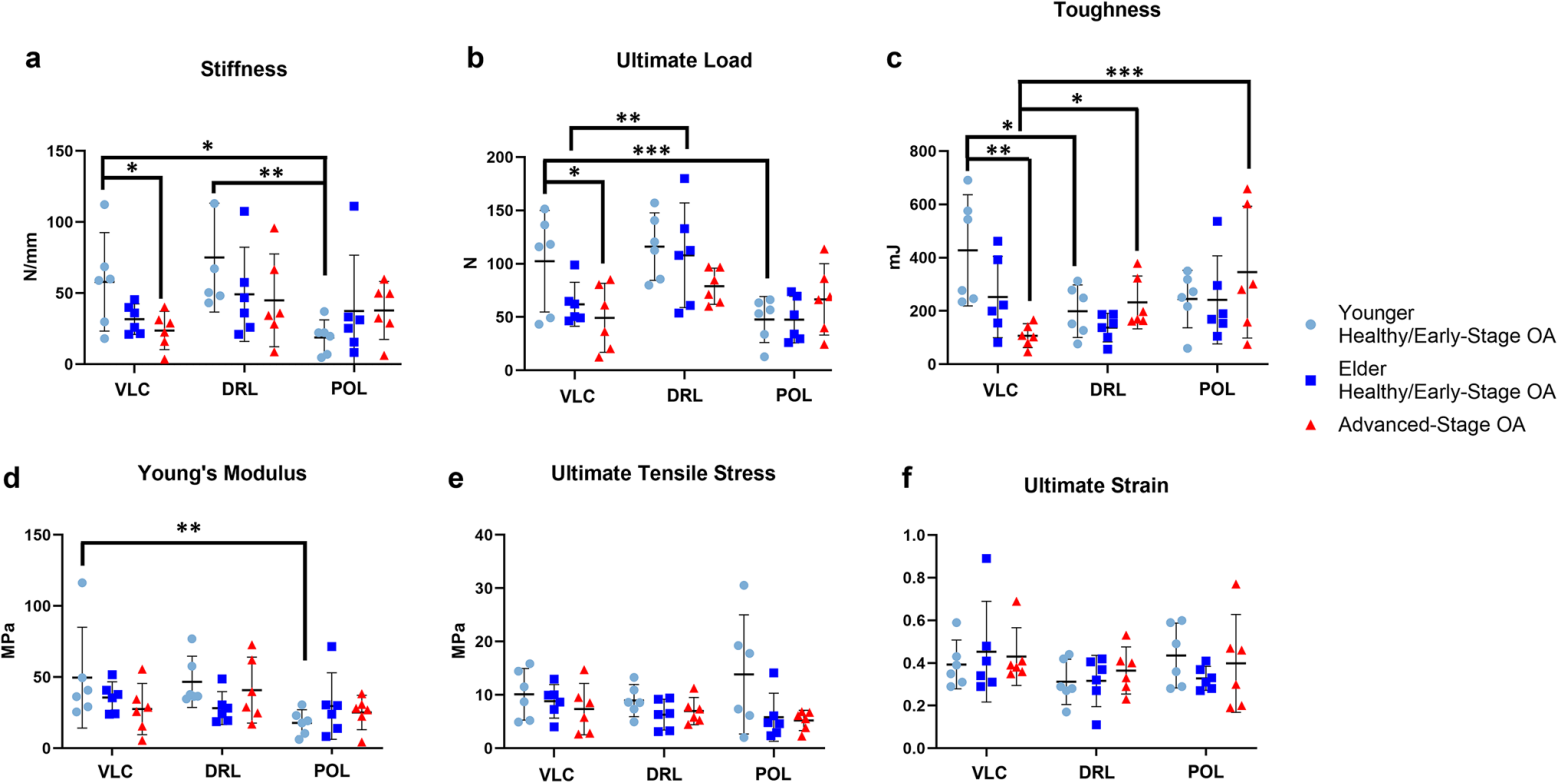
Load-to-failure results: (**a**) stiffness, (**b**) ultimate load, (**c**) toughness, (**d**) Young’s modulus, (**e**) ultimate tensile stress, and (**f**) ultimate strain. Data shown are mean ± 95% confidence interval. *P < 0.05, **P < 0.01, ***P < 0.001

Load-to-failure outcomes further demonstrated a decrease of VLC stiffness (57.94 ± 33.04 N/mm to 23.73 ± 12.82 N/mm, *P* = 0.048), ultimate load (102.41 ± 45.42 N to 49.28 ± 30.85 N, *P* = 0.065), and toughness (427.91 ± 198.83 mJ to 107.29 ± 42.75 mJ, *P* = 0.003) in the advanced-stage OA group when compared to younger healthy/early-stage OA group (Fig. 6). Additionally, load-to-failure outcomes demonstrated that, in the younger healthy/early-stage OA group, VLC had a higher toughness (427.91 ± 198.83 mJ) than that of DRL (199.25 ± 94.01 mJ, *P* = 0.035) and higher stiffness (VLC: 57.94 ± 33.04 N/mm, POL: 18.78 ± 11.80 N/mm, *P* = 0.043), ultimate load (VLC: 102.41 ± 45.42 N, POL: 47.68 + 20.58 N, *P* = 0.001) and Young’s modulus (VLC: 49.64 ± 33.78 MPa, POL: 17.86 + 8.74 MPa, *P* = 0.014) than that of POL (Fig. 6). DRL was found to have a higher ultimate load (107.99 ± 46.82 N) than VLC (61.98 + 19.58 N, *P* = 0.006) and POL (47.54 ± 20.58 N, *P* < 0.001) in the elder healthy/early-stage OA group. Additionally, VLC had a lower toughness (107.29 ± 42.75 mJ) than that of DRL (232.18 ± 94.34 mJ, *P* = 0.043) and POL (345.63 ± 235.87 mJ, P < 0.001) in the advanced-stage OA group. Sample mean and standard deviation was listed in Tables 3 and 4 in Online Resource 1. Detailed model-based comparisons of stress relaxation and load-to-failure outcomes were listed in Tables 7, 8, 9, and 10 in Online Resource 1.

### Failure Patterns Under Tension

**Fig. 7:**
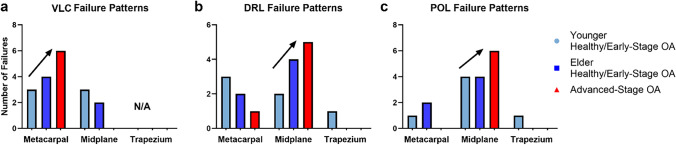
Local strain patterns: (**a**) location of failures for VLC; upwards trend of failures in metacarpal as disease progressed, (**b**) location of failures for DRL; upwards trend of failures in midplane as disease progressed, and (**c**) location of failures for POL; upwards trend of failures in midplane as disease progressed

Load-to-failure tensile experiments showed that VLC was more likely to fail at the metacarpal insertion as OA advanced [younger healthy/early-stage OA: metacarpal insertion (3/6 specimens), midplane (3/6); elder healthy/early-stage OA: metacarpal insertion (4/6), midplane (2/6); advanced OA: all at metacarpal insertion (6/6)] (Fig. 7a). No VLC failed at the trapezium insertion in any OA group. By contrast, DRL failure site trended towards the midplane as OA progressed [younger healthy/early-stage OA: midplane (2/6), metacarpal insertion (3/6), trapezium insertion (1/6); elder healthy/early-stage OA: midplane (4/6), metacarpal insertion (2/6); advanced OA: all at midplane (6/6)] (Fig. 7b). Similar trend of failure towards the midplane was found for the POL [younger healthy/early-stage OA: midplane (4/6), metacarpal insertion (1/6), trapezium insertion (1/6); elder healthy/early-stage OA: midplane (4/6), metacarpal insertion (2/6); advanced-stage OA, all at midplane (6/6)] (Fig. 7c). The failure patterns of VLC, DRL, and POL were further validated by the local strain analysis, where strain concentration was found to be highest near the failure site (Fig. 1, Online Resource 2).

### Structural Integrity and Mineral Distribution

**Fig. 8:**
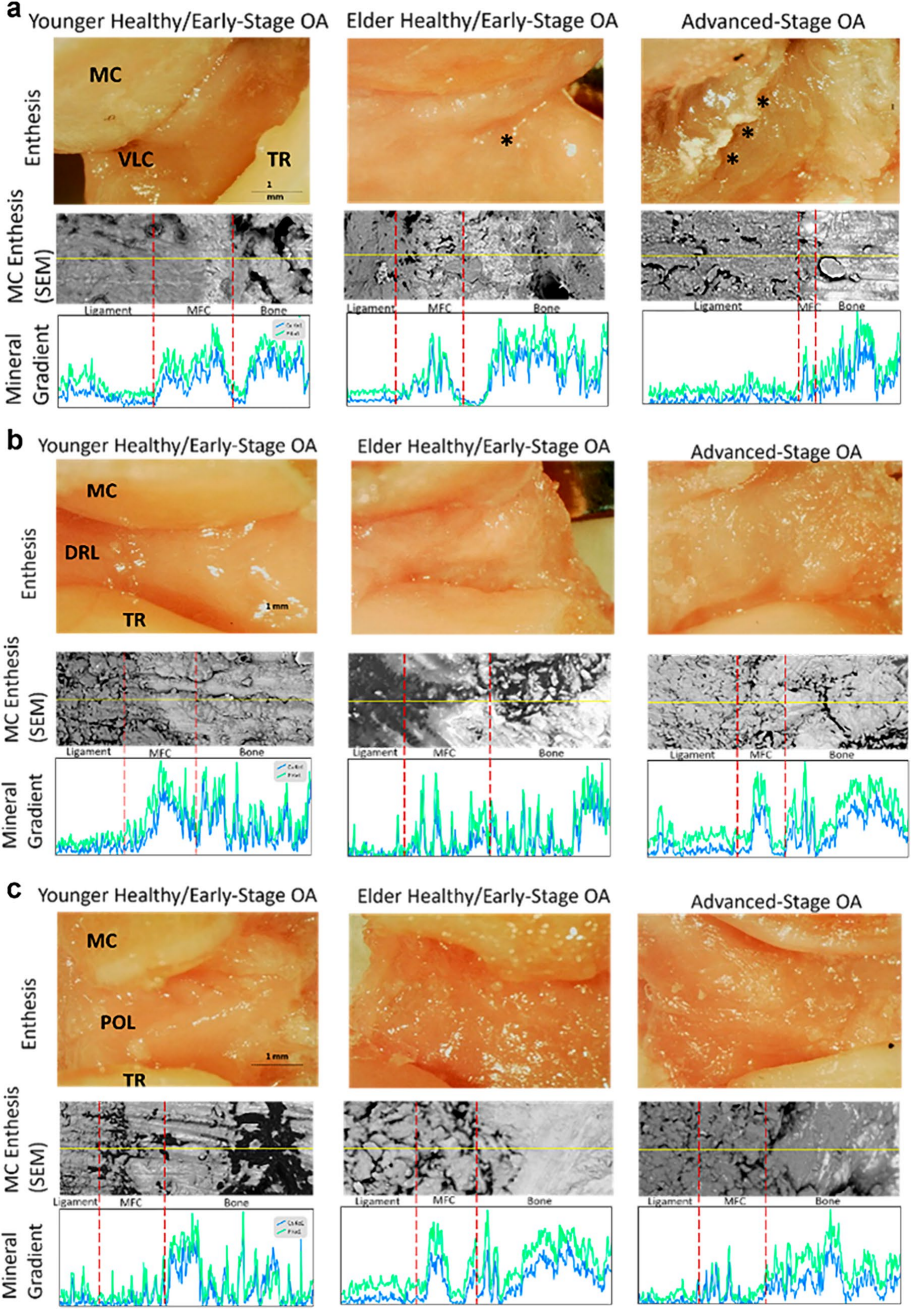
Light microscopy, SEM imaging, and EDS analysis of metacarpal insertion of (**a**) VLC, (**b**) DRL, (**c**) POL. * denotes beginning of detachment at MC enthesis. *** denotes detachment and calcification at MC enthesis. Blue represents general levels of calcium, and green represents general levels of phosphorus. MFC stands for mineralized fibrocartilage

Light microscopy imaging revealed that the VLC partially detached from the metacarpal beak adjacent to the BL insertion in the elder healthy/early-stage OA group. Aberrant calcification and further ligament deterioration were observed around the VLC metacarpal enthesis in the advanced-stage OA group. By contrast, no clear detachment or degeneration was seen in the DRL and POL insertions across all OA groups. SEM and EDS analysis further revealed a loss of smooth transitional mineral gradients across the VLC metacarpal enthesis during OA progression (Fig. 8a). Specifically, in the younger healthy/early-stage OA group, levels of calcium and phosphate began at a low point in the ligament portion, gradually increasing within the mineralized fibrocartilage portion of the enthesis, and eventually reaching their highest level in the bone. This smooth transition is indicated by the broad peaks observed in the EDS linescan, spanning a significant portion of the enthesis, and connecting non-mineralized ligament to fully ossified bone (Fig. 8a). This fibrocartilaginous transition becomes less prominent, but still recognizable, at the metacarpal enthesis of the VLCs in elder healthy/early-stage OA TMC joints. The metacarpal enthesis of VLCs in the advanced-stage OA TMC joints exhibited a much steeper mineral gradient, with calcium and phosphate levels starting low in the ligament, then abruptly spiking as bone was approached, lacking the broad peaks that typically represent the fibrocartilaginous transition. No distinct changes in the mineral gradient transition zones were observed at the DRL and POL metacarpal entheses (Fig. 8b, c) or at the trapezium entheses of all three ligaments (Fig. 2a–c, Online Resource 2).

## Discussion

This study addresses the knowledge gap concerning the morphological and mechanical changes TMC ligaments undergo during OA progression. From the results of this study, we concluded that mechanical properties of the VLC in advanced-stage osteoarthritic TMCs attenuate compared to healthy/early-stage OA groups, as evidenced by moduli in stress relaxation, as well as stiffness, ultimate load, and toughness in load-to-failure tests. These attenuated structures likely lead to joint instability resulting in an increase in volar-dorsal and ulnar-radial metacarpal translation [14]. Previous research has found that contact patterns of the joint are often concentrated in the palmar-ulnar region and spreads dorsoradially as OA progresses, thus supporting the idea that increased metacarpal translation is occurring during OA progression. These contact patterns further correlated with areas of increased articular cartilage wear [8, 15, 16, 23]. Unlike the attenuation of VLC mechanical properties, DRL and POL displayed no mechanical changes throughout OA and age progression. Therefore, they could potentially contribute to stabilizing the dorsal side of the TMC joint. This is supported by a TMC articular cartilage wear study that states that the dorsal compartment is the only area that consistently has healthy cartilage in diseased joints [16].

The overall attenuated mechanical properties and increased failure rate at the metacarpal insertion seen in VLCs of advanced-stage osteoarthritic TMC joints can be attributed to partial detachment of the deep intraarticular layers of collagen fiber bundles in the BL, loss of the transitional mineral gradient, and aberrant calcification buildup around the metacarpal insertion of advanced-stage osteoarthritic joints, as observed through light microscopy and SEM imaging. Previous cadaveric histological studies have documented the degeneration and detachment of intraarticular BL fibrocartilage [8, 14, 16]. As acknowledged by previous soft tissue-bone interface research, healthy entheses retain a gradient in mechanical, structural, and compositional properties to aid in the distribution of force [24–26]. Specifically, studies conducted on the entheses of supraspinatus tendon, rotator cuff, and anterior cruciate ligament have shown a smooth transition of collagen structure and mineralization from soft tissue to bone, as opposed to an abrupt interface between the two [24, 25, 27–29]. Consequently, the absence of a smooth mineral gradient combined with aberrant buildup of calcification observed at the metacarpal enthesis of VLCs in advanced-stage osteoarthritic TMC joints, along with intraarticular collagen bundle partial detachment, could result in increased stress concentrations at the ligamentous attachment. This could lead to premature failure of the diseased ligament under tension. Our local strain analysis further supported this idea, indicating the presence of stress and strain concentrations at the metacarpal enthesis of the VLC in advanced-stage osteoarthritic TMC joints, likely contributing to enthesis failure under tension. Additionally, no structural or mineral change was seen at either the metacarpal or trapezium insertions for the DRL and POL in the different OA groups, further supporting their lack of change in mechanical properties and their tendency to fail at the midplane as OA progressed.

An interesting age-related observation emerges when comparing the stress relaxation and load-to-failure results. In the stress relaxation experiments, significant changes are observed in VLCs between the elder healthy/early-stage OA group and advanced-stage OA group. This contrasts with the load-to-failure results, where the most significant changes in VLCs are seen between the younger healthy/early-stage OA group and advanced-stage OA group. This may be due to the combination of age-related factors and calcification associated with disease progression. Our light microscopy and SEM/EDS data reveal that the elder group exhibited early VLC (specifically BL) detachment, mineral gradient degradation, and aberrant calcification, while these changes were not present in the younger group. These observations, which are not detectable using the radiographic Eaton-Littler scale, suggest that elder samples had a more advanced form of TMC OA than indicated by their diagnoses. In the stress relaxation tests, the low, gradually increasing strain increments, combined with the early stages of mineral gradient degeneration and calcification build-up may have improved the ligament’s response in the elder healthy/early-stage group, leading to the greatest difference being between this group and the advanced-stage OA group. Conversely, in the load-to-failure tests, where mineral gradient loss and calcification are coupled with high strain levels and increased strain rate, premature failure is observed in the elderly healthy/early-stage group [26]. Here, the largest differences are seen between the younger healthy/early-stage OA group and the advanced-stage OA group.

Notably, among the ligaments investigated in this study, the VLC was the only one to exhibit a significant form of change as both OA and age progressed, specifically at its intracapsular metacarpal insertion. This leads us to consider the degeneration and detachment of the metacarpal insertion of the VLC as a potential “tipping point” for TMC OA pathomechanics. Previous studies have highlighted the mechanical significance of the DRL and POL as stabilizers of the TMC joint [6, 9, 30, 31], and the results found in this study do support this concept by showing that there is no significant change in the properties of DRL and POL during OA progression. We postulate that these ligaments, alongside osteophyte formation and overall flattening of bony morphometry, collectively play a crucial role in joint stabilization following the loss of mechanical and structural integrity in the VLC. Additionally, the DRL and POL have been found to possess an increased presence of mechanoreceptors and nerve endings compared to the VLC [32], suggesting their ability to sense joint instability, particularly at the onset of TMC OA when the VLC begins to degenerate or partially detach under tension.

Previous research has generally found the DRL to exhibit superior mechanical properties in terms of toughness, ultimate load, and stiffness compared to the ligaments within the VLC [6, 10, 11]. However, our results differ from these findings as we observed similar magnitude of mechanical properties of the VLC, DRL, and POL in healthy/early-stage specimens. This discrepancy can be attributed to our approach of utilizing three distinct groups of female VLCs in our study, as described in Table 1 of Online Resource 1, rather than combining them into a single group regardless of age and OA stage. In addition to comparing healthy and osteoarthritic TMC joints, we further incorporated aging as a factor by dividing the healthy/early-stage OA specimens into younger and elder subsets with the cutoff age set at 45 (the average age of perimenopause) [33]. Another distinctive aspect of this study compared to previous ones is the incorporation of stress relaxation experiments. While previous studies have focused on the TMC ligaments’ response to load-to-failure tests, to our knowledge, none have investigated their response to stress relaxation. Conducting stress relaxation experiments is beneficial because they provide a closer examination of the ligaments’ mechanical behavior within the initial toe region under tension and part of the elastic region at lower strain levels. This approach offers additional insights into the intrinsic tensile material properties of the ligaments.

## Limitations and Future Work

Limitations of this study include the use of the Eaton-Littler classification system to grade the hand specimens’ OA severity. Despite being graded by multiple orthopaedic surgeons, this system solely relies on radiographic evaluation of articulating surfaces, which demonstrates limited inter- and intra-observer reliability [34–37]. Furthermore, the stress relaxation experiments revealed some variability in the mechanical moduli among the younger healthy/early-stage osteoarthritic subjects. This variability may be attributed to some samples exhibiting higher moduli due to onset of aberrant calcification around the ligament insertion, similar to that observed in the elder healthy/early-stage osteoarthritic group. However, these subjects cannot be distinguished from the elder healthy/early-stage osteoarthritic group based on either the radiographic evidence of articulating surfaces using the Eaton-Littler scale, or the cutoff age set at 45. Variation could also stem from human error when loading specimens into the grips of the testing apparatus. Care was taken to assure proper fiber alignment with the grips, but any slight difference in loading position from sample to sample could cause variance in the mechanical testing results. In order to more easily make comparisons between ligament types and disease group, the relaxation periods between each strain increment were kept constant for each ligament. As a result, some ligaments may not have reached full equilibrium, especially at the 20% strain level, which could also be a cause for variation. Another limitation is the exclusive use of female specimens in this study. The goal was to focus on how disease progression and aging affect the morphological and mechanical properties of TMC ligaments in females, who are the most predisposed population to TMC OA. Future studies will include specific dissection and testing of the sAOL and BL as discrete components of the VLC, as well as increasing sample sizes and incorporating male cohorts to explore sexual dimorphism in TMC pathomechanics.

## Conclusion

In conclusion, the VLC, including the sAOL and BL, experiences attenuated mechanical properties in advanced-stage osteoarthritic TMCs which may be associated with intraarticular ligament detachment and degradation, as well as the loss of a smooth mineral transition at the metacarpal enthesis. By contrast, the DRL and POL mechanical, structural, and mineral composition properties remain unaltered through OA progression.

## Supplementary Information

Below is the link to the electronic supplementary material.

Supplementary file1 (PDF 1165 KB)
