# Correction: Morphological and Mechanical Property Differences in Trapeziometacarpal Ligaments of Healthy and Osteoarthritic Female Joints

**DOI:** 10.1007/s10439-025-03694-2

**Published:** 2025-02-14

**Authors:** Lizzie Walker, Daniel Gordon, Alexander Chiaramonti, Shangping Wang, Zhaoxu Meng, Dane Daley, Elizabeth Slate, Hai Yao, Vincent D. Pellegrini, Yongren Wu

**Affiliations:** 1 Department of Bioengineering, Clemson University, 68 President Street, BEB 203, Charleston, SC 29425 USA; 2 Department of Orthopaedic Surgery and Rehabilitation, Wake Forest University School of Medicine, Winston Salem, NC USA; 3 Department of Orthopaedics and Physical Medicine, Medical University of South Carolina, Charleston, SC USA; 4 Department of Mechanical Engineering, Clemson University, Clemson, SC USA; 5 Department of Statistics, Florida State University, Tallahassee, FL USA; 6 Department of Orthopaedics, Dartmouth-Hitchcock Medical Center, Lebanon, NH USA

**Correction to: Annals of Biomedical Engineering (2024)** 10.1007/s10439-024-03660-4

The original article has been updated to correct multiple errors in the units of measure displayed in the section “Tensile Mechanical Properties”.

