# Supplementary material for: Morphological and Mechanical Property Differences in Trapeziometacarpal Ligaments of Healthy and Osteoarthritic Female Joints
Source: Ann Biomed Eng. 2024 Dec 7;53(4):799–811. doi: 10.1007/s10439-024-03660-4 (PMC11929737; doi:10.1007/s10439-024-03660-4)
Supplement: Supplementary file 1 — Supplementary file1 (PDF 1165 KB) [file 10439_2024_3660_MOESM1_ESM.pdf]

Online Resource 1
Morphological and Mechanical Property Differences in Trapeziometacarpal Ligaments of Healthy and Osteoarthritic Female Joints

Lizzie Walker[1] (mewlkr@g.clemson.edu, ORCiD: 0009-0001-9980-6260), Daniel Gordon[1] (digordo@g.clemson.edu), Alexander Chiaramonti[2] (achiaram@wakehealth.edu), Shangping Wang[1,3] (shagpw@clemson.edu ORCiD: 0000-0003-0049-2212), Zhaoxu Meng[4] (zmeng@clemson.edu ORCiD: 0000-0002-3250-7696), Dane Daley[3] (dalda@musc.edu), Elizabeth Slate[5] (eslate@fsu.edu), Hai Yao[1,3] (haiyao@clemson.edu), Vincent D. Pellegrini, Jr. (vincent.d.pellegrini.jr@dartmouth.edu) [6], Yongren Wu[1,3] (yongren@clemson.edu ORCiD: 0000-0002-5411-8528)

Author Affiliations:
(1) Department of Bioengineering, Clemson University, Clemson, SC
(2) Department of Orthopaedic Surgery and Rehabilitation, Wake Forest University School of Medicine, Winston Salem, NC
(3) Department of Orthopaedics and Physical Medicine, Medical University of South Carolina, Charleston, SC
(4) Department of Mechanical Engineering, Clemson University, Clemson, SC
(5) Department of Statistics, Florida State University, Tallahassee, FL
(6) Department of Orthopaedics, Dartmouth-Hitchcock Medical Center, Lebanon, NH

Corresponding Author: Yongren Wu, yongren@clemson.edu, 843-876-2305, 68 President Street, BEB 203, Charleston, SC, 29425

Specimen ID	Age	Grade	Race	Hand
**Younger Healthy/Early-Stage OA**				
1	28	0	C	L
2	42	II	C	L
3	42	0	C	R
4	42	I	C	L
5	44	II	C	R
6	42	I	C	L
7	41	I	C	L
8	45	I	C	R
**Elder Healthy/Early-Stage OA**				
9	55	0	C	L
10	65	II	C	L
11	66	II	C	R
12	64	I	B	R
13	73	II	C	L
14	73	II	C	R
15	56	I	C	R
16	59	II	C	L
**Advanced-Stage OA**				
17	66	IV	C	L
18	72	IV	C	L
19	65	III	B	R
20	72	III	C	R
21	59	III	C	L
22	53	III	C	R
23	38	III	C	R
24	53	III	C	L

Supplemental Table 1. Age, grade, race (C = Caucasian, B = black), and hand (R = right, L = left) for each specimen

		Width (mm)	Thickness (mm)	Length (mm)
Younger Healthy/Early-Stage OA n = 6	VLC	7.5 ± 2.28	1.44 ± 0.37	8.38 ± 0.95
	DRL	7.26 ± 1.41	2.16 ± 0.51	9.21 ± 1.91
	POL	5.76 ± 1.05	1.90 ± 0.36	9.91 ± 2.20
Elder Healthy/Early-Stage OA n = 6				
	VLC	7.06 ± 1.69	1.38 ± 0.52	8.33 ± 1.96
	DRL	7.58 ± 1.63	2.37 ± 0.51	9.63 ± 2.53
	POL	6.32 ± 1.44	1.87 ± 0.49	9.67 ± 2.94
Advanced-Stage OA n = 6				
	VLC	5.25 ± 0.52	1.20 ± 0.36	7.49 ± 1.09
	DRL	6.55 ± 1.40	2.05 ± 0.30	8.46 ± 1.71
	POL	6.58 ± 0.19	1.94 ± 0.39	8.49 ± 0.86

Supplemental Table 2. Ligament dimensions; represented as sample mean ± sample standard deviation

		Young's Modulus (MPa)				Instantaneous Modulus (MPa)	Relaxed Modulus (MPa)
		5%	10%	15%	20%		
Younger Healthy/Early-Stage OA n = 6	VLC	7.70 ± 5.02	11.83 ± 7.08	15.96 ± 9.12	19.79 ± 9.92	8.56 ± 3.71	5.72 ± 3.22
	DRL	6.75 ± 5.18	9.85 ± 6.91	13.74 ± 7.17	17.66 ± 7.89	8.38 ± 5.03	5.64 ± 3.70
	POL	5.92 ± 3.61	9.94 ± 5.78	15.04 ± 6.39	21.26 ± 10.96	8.30 ± 3.54	5.46 ± 2.73
Elder Healthy/Early-Stage OA n = 6							
	VLC	7.76 ± 1.57	14.25 ± 3.17	20.57 ± 3.47	26.36 ± 4.20	12.55 ± 1.19	8.89 ± 1.48
	DRL	4.64 ± 3.36	10.3 ± 7.77	14.35 ± 9.78	17.60 ± 8.69	7.03 ± 4.17	4.51 ± 3.13
	POL	5.68 ± 3.76	9.80 ± 5.10	15.19 ± 7.93	16.16 ± 5.33	7.86 ± 2.67	5.02 ± 2.15
Advanced-Stage OA n = 6							
	VLC	5.05 ± 2.22	7.44 ± 3.63	10.89 ± 4.55	14.68 ± 5.59	7.11 ± 1.97	4.83 ± 1.34
	DRL	4.14 ± 1.85	8.21 ± 4.05	14.61 ± 7.70	18.04 ± 8.78	8.36 ± 3.83	5.38 ± 2.68
	POL	5.63 ± 2.01	9.97 ± 3.98	14.15 ± 5.40	17.69 ± 7.50	7.70 ± 3.40	5.17 ± 2.45

Supplemental Table 3. Stress-relaxation results; represented as sample mean ± sample standard deviation

		Young's Modulus (MPa)	Ultimate Tensile Stress (MPa)	Ultimate Strain	Stiffness(N/mm)	Ultimate Load (N)	Toughness (Nmm)
Younger Healthy/Early-Stage OA n = 6	VLC	49.64 ± 33.78	10.11 ± 4.61	0.39 ± 0.11	57.94 ± 33.04	102.41 ± 45.42	427.91 ± 198.83
	DRL	46.68 ± 17.12	8.95 ± 2.88	0.31 ± 0.10	75.04 ± 36.52	116.05 ± 30.18	199.25 ± 94.01
	POL	17.86 ± 8.74	4.62 ± 2.11	0.44 ± 0.14	18.78 ± 11.80	47.68 ± 20.58	244.7 ± 102.44
Elder Healthy/Early-Stage OA n = 6							
	VLC	35.63 ± 10.58	8.81 ± 3.00	0.5 ± 0.34	31.68 ± 10.26	61.98 ± 19.58	252.10 ± 145.42
	DRL	28.24 ± 11.03	6.30 ± 2.74	0.32 ± 0.12	57.50 ± 25.93	107.99 ± 46.82	137.42 ± 51.15
	POL	29.66 ± 22.31	5.82 ± 4.29	0.33 ± 0.05	37.47 ± 37.36	47.54 ± 20.77	241.53 ± 157.62
Advanced-Stage OA n = 6							
	VLC	27.62 ± 17.14	7.33 ± 4.60	0.43 ± 0.13	23.73 ± 12.82	49.28 ± 30.85	107.29 ± 42.75
	DRL	40.87 ± 22.10	6.98 ± 2.43	0.37 ± 0.11	44.77 ± 30.98	78.93 ± 16.22	232.18 ± 94.34
	POL	25.15 ± 11.46	5.22 ± 1.81	0.40 ± 0.22	37.80 ± 19.38	66.56 ± 31.97	345.62 ± 235.87

Supplemental Table 4. Load-to-failure results; represented as sample mean ± sample standard deviation

	VLC YH/VLC EH	VLC YH/VLC OA	VLC EH/VLC OA	DRL YH/DRL EH	DRL YH/DRL OA	DRL EH/DRL OA	POL YH/POL EH	POL YH/POL OA	POL EH/POL OA
	p. adj	*p. adj*	*p. adj*	*p. adj*	*p. adj*	*p. adj*	*p. adj*	*p. adj*	*p. adj*
Width	1	**0.009***	**0.034***	1	1	0.447	1	0.845	1
Thickness	1	0.935	1	1	1	0.480	1	1	1
Length	1	1	1	1	1	0.945	1	1	1
	Mean diff (95% CI)	*Mean diff (95% CI)*	*Mean diff (95% CI)*	*Mean diff (95% CI)*	*Mean diff (95% CI)*	*Mean diff (95% CI)*	*Mean diff (95% CI)*	*Mean diff (95% CI)*	*Mean diff (95% CI)*
Width	0.445 (-1.253, 2.143)	2.239 (0.449, 4.028)	1.794 (0.101, 3.486)	-0.310 (-2.008, 1.388)	0.696 (-1.094, 2.486)	1.006 (-0.686, 2.699)	-0.574 (-2.313, 1.165)	-0.854 (-2.786, 1.078)	-0.280 (-2.158, 1.599)
Thickness	0.078 (-0.435, 0.590)	0.223 (-0.316, 0.762)	0.146 (-0.354, 0.646)	-0.193 (-0.705, 0.319)	0.096 (-0.443, 0.635)	0.289 (-0.211, 0.789)	0.073 (-0.450, 0.596)	-0.047 (-0.624, 0.530)	-0.120 (-0.670, 0.430)
Length	0.154 (-2.144, 2.452)	0.633 (-1.757, 3.023)	0.479 (-1.484, 2.442)	-0.315 (-2.613, 1.983)	0.494 (-1.896, 2.884)	0.809 (-1.154, 2.772)	0.429 (-1.892, 2.751)	0.904 (-1.568, 3.377)	0.475 (-1.605, 2.554)

Supplemental Table 5. Comparison of ligament dimensions between disease groups derived from linear mixed effects model

	VLC YH/DRL YH	VLC YH/POL YH	DRL YH/POL YH	VLC EH/DRL EH	VLC EH/POL EH	DRL EH/POL EH	VLC OA/DRL OA	VLC OA/POL OA	DRL OA/POL OA
	p. adj	*p. adj*	*p. adj*	*p. adj*	*p. adj*	*p. adj*	*p. adj*	*p. adj*	*p. adj*
Width	1	0.54	0.119	1	0.818	0.191	0.218	0.258	1
Thickness	**0.002***	0.069	0.512	**<0.001***	**0.040***	**0.013***	**<0.001***	**0.004***	1
Length	0.504	**0.041***	0.750	0.056	0.085	1	0.328	0.189	1
	Mean diff (95% CI)	*Mean diff (95% CI)*	*Mean diff (95% CI)*	*Mean diff (95% CI)*	*Mean diff (95% CI)*	*Mean diff (95% CI)*	*Mean diff (95% CI)*	*Mean diff (95% CI)*	*Mean diff (95% CI)*
Width	0.237 (-1.531, 2.006)	1.744 (-0.024, 3.512)	1.506 (-0.262, 3.274)	-0.517 (-2.099, 1.064)	0.725 (-0.900, 2.350)	1.242 (-0.382, 2.867)	-1.305 (-3.073, 0.463)	-1.348 (-3.258, 0.561)	-0.043 (-1.953, 1.866)
Thickness	-0.720 (-1.198, -0.242)	-0.452 (-0.931, 0.026)	0.268 (-0.211, 0.746)	-0.991 (-1.419, -0.563)	-0.457 (-0.898, -0.016)	0.534 (0.092, 0.975)	-0.847 (-1.300, -0.394)	-0.723 (-1.245, -0.201)	0.124 (-0.398, 0.646)
Length	-0.837 (-2.324, 0.649)	-1.534 (-3.021, -0.047)	-0.696 (-2.183, 0.791)	-1.306 (-2.636, 0.024)	-1.258 (-2.638, 0.122)	0.048 (-1.332, 1.428)	-0.976 (-2.463, 0.511)	-1.263 (-2.910, 0.385)	-0.286 (-1.934, 1.361)

Supplemental Table 6. Comparison of ligament dimensions between ligament types derived from linear mixed effects model

	VLC YH/VLC EH	VLC YH/VLC OA	VLC EH/VLC OA	DRL YH/DRL EH	DRL YH/DRL OA	DRL EH/DRL OA	POL YH/POL EH	POL YH/POL OA	POL EH/POL OA
	p. adj	*p. adj*	*p. adj*	*p. adj*	*p. adj*	*p. adj*	*p. adj*	*p. adj*	*p. adj*
Young's 5%	1	0.379	0.552	0.169	0.135	1	1	1	1
Young's 10%	1	0.448	**0.104**	0.868	0.468	1	1	1	1
Young's 15%	0.916	0.694	**0.088**	0.212	0.464	1	1	1	1
Young's 20%	0.546	0.729	**0.044***	0.068	0.534	1	1	1	1
Instantaneous	0.193	1	**0.023***	0.253	0.891	1	1	1	1
Relaxed	0.149	1	**0.017***	0.085	0.579	1	1	1	1
	Mean diff (95% CI)	*Mean diff (95% CI)*	*Mean diff (95% CI)*	*Mean diff (95% CI)*	*Mean diff (95% CI)*	*Mean diff (95% CI)*	*Mean diff (95% CI)*	*Mean diff (95% CI)*	*Mean diff (95% CI)*
Young's 5%	0.411 (-4.382, 5.205)	3.019 (-1.187, 7.854)	2.607 (-2.218, 7.433)	3.663 (-0.996, 8.323)	3.996 (-0.833, 8.826)	0.333 (-1.366, 5.032)	0.745 (-3.927, 5.417)	0.872 (-3.957, 5.702)	0.127 (-4.584, 4.839)
Young's 10%	-2.297 (-10.320, 5.727)	4.743 (-3.298, 12.784)	7.040 (-0.992, 15.072)	3.340 (-4.405, 11.086)	4.661 (-3.373, 12.696)	1.321 (-6.440, 9.082)	0.555 (-7.2, 8.311)	0.579 (-7.455, 8.614)	0.024 (-7.747, 7.795)
Young's 15%	-4.780 (-16.236, 6.675)	5.595 (-5.862, 17.052)	10.375 (-1.080, 21.831)	8.229 (-2.811, 19.269)	6.676 (-4.780, 18.132)	-1.553 (-12.594, 9.488)	0.051 (-10.990, 11.092)	1.146 (-10.310, 12.602)	1.095 (-9.948, 12.137)
Young's 20%	-6.600 (-18.698, 5.499)	5.761 (-6.339, 17.861)	12.361 (0.262, 24.460)	8.163 (-3.497, 19.822)	6.351 (-6.339, 17.861)	-1.812 (-13.437, 9.849)	5.162 (-6.499, 16.822)	3.674 (-8.425, 15.773)	-1.488 (-13.150, 10.174)
Instantaneous	-3.954 (-9.135, 1.227)	1.879 (-3.307, 7.065)	5.833 (0.650, 11.016)	4.742 (-0.254, 9.738)	2.853 (-2.331, 8.037)	-1.889 (0.650, 11.016)	0.485 (-4.515, 5.485)	0.899 (-4.285, 6.082)	0.414 (-4.591, 5.419)
Relaxed	-3.103 (-6.931, 0.725)	1.359 (-2.473, 5.192)	4.462 (0.633, 8.292)	3.366 (-0.326, 7.058)	2.035 (-1.795, 5.866)	-1.330 (-5.027, 2.366)	0.514 (-3.181, 4.209)	0.602 (-3.228, 4.433)	0.088 (-3.611, 3.788)

Supplemental Table 7. Comparison of stress relaxation experiments between disease group derived from linear mixed effects model

	VLC YH/DRL YH	VLC YH/POL YH	DRL YH/POL YH	VLC EH/DRL EH	VLC EH/POL EH	DRL EH/POL EH	VLC OA/DRL OA	VLC OA/POL OA	DRL OA/POL OA
	p. adj	*p. adj*	*p. adj*	*p. adj*	*p. adj*	*p. adj*	*p. adj*	*p. adj*	*p. adj*
Young's 5%	1	0.988	0.854	0.116	0.614	1	1	1	0.932
Young's 10%	1	1	1	0.259	0.358	1	1	1	1
Young's 15%	0.806	1	0.577	0.222	0.569	1	1	1	1
Young's 20%	1	1	1	**0.089**	**0.089**	1	1	1	1
Instantaneous	0.656	1	0.548	**0.006***	**0.05***	1	1	1	1
Relaxed	0.937	1	0.715	**0.003***	**0.025***	1	1	1	1
	Mean diff (95% CI)	*Mean diff (95% CI)*	*Mean diff (95% CI)*	*Mean diff (95% CI)*	*Mean diff (95% CI)*	*Mean diff (95% CI)*	*Mean diff (95% CI)*	*Mean diff (95% CI)*	*Mean diff (95% CI)*
Young's 5%	-0.200 (-7.959, 6.485)	1.439 (-5.710, 8.733)	1.640 (-5.080, 9.577)	3.051 (-0.527, 6.630)	1.773 (-1.706, 5.251)	-1.314 (-5.623, 2.995)	0.777 (-2.883, 4.437)	-0.707 (-4.367, 2.953)	-1.485 (-5.145, 2.175)
Young's 10%	-0.737 (-12.556, 9.526)	1.512 (-9.151, 12.931)	2.249 (-7.636, 14.446)	4.900 (-2.093, 11.893)	4.363 (-2.541, 11.268)	-0.537 (-7.209, 6.136)	-0.819 (-8.033, 6.395)	-2.652 (-9.866, 4.562)	-1.834 (-9.048, 5.381)
Young's 15%	-5.040 (-16.337, 6.257)	0.942 (-10.355, 12.239)	5.982 (-5.316, 17.281)	7.969 (-2.917, 18.856)	5.773 (-5.119, 16.666)	-2.196 (-12.656, 8.265)	-3.959 (-15.259, 7.341)	-3.507 (-14.807, 7.793)	0.452 (-10.848, 11.752)
Young's 20%	-4.341 (-16.341, 7.660)	-1.325 (-13.325, 10.675)	3.016 (-8.970, 15.001)	10.422 (-1.137, 21.980)	10.436 (-1.144, 22.016)	0.015 (-11.098, 11.128)	-3.751 (-15.755, 8.253)	-3.412 (-15.416, 8.592)	0.339 (-11.665, 12.343)
Instantaneous	-2.414 (-7.284, 2.456)	0.238 (-4.632, 5.107)	2.652 (-2.254, 7.558)	6.282 (1.577, 10.987)	4.677 (.000, 9.354)	-1.605 (-6.109, 2.899)	-1.440 (-6.310, 3.430)	-0.742 (-5.612, 4.128)	0.697 (-4.173, 5.568)
Relaxed	-1.457 (-5.073, 2.159)	0.270 (-3.346, 3.886)	1.727 (-1.907, 5.361)	5.012 (1.520, 8.503)	3.887 (0.408, 7.366)	-1.125 (-4.470, 2.221)	-0.781 (-4.399, 2.837)	-0.487 (-4.105, 3.131)	0.294 (-3.324, 3.912)

Supplemental Table 8. Comparison of stress relaxation experiments between ligament types derived from linear mixed effects model

	VLC YH/VLC EH	VLC YH/VLC OA	VLC EH/VLC OA	DRL YH/DRL EH	DRL YH/DRL OA	DRL EH/DRL OA	POL YH/POL EH	POL YH/POL OA	POL EH/POL OA
	p. adj	*p. adj*	*p. adj*	*p. adj*	*p. adj*	*p. adj*	*p. adj*	*p. adj*	*p. adj*
Stiffness	0.262	**0.048***	1	0.767	0.16	1	0.691	0.676	1
Ult. Load	0.062	**0.017***	1	1	0.164	0.509	1	0.902	0.774
Toughness	0.204	**0.003***	0.268	1	1	0.299	1	0.054	0.06
Young's	0.537	0.148	1	0.296	1	0.722	0.868	1	1
Ult. Tensile Stress	1	0.523	1	0.443	1	1	1	1	1
Ult. Strain	0.773	1	1	1	1	1	0.84	1	1
	Mean diff (95% CI)	*Mean diff (95% CI)*	*Mean diff (95% CI)*	*Mean diff (95% CI)*	*Mean diff (95% CI)*	*Mean diff (95% CI)*	*Mean diff (95% CI)*	*Mean diff (95% CI)*	*Mean diff (95% CI)*
Stiffness	26.562 (-11.234, 64.358)	37.272 (0.230, 74.314)	7.462 (-30.331, 45.255)	17.503 (-20.294, 55.301)	30.163 (-7.633, 67.959)	12.660 (-25.136, 50.455)	-18.488 (-56.285, 19.310)	-18.687 (-56.483, 19.108)	-0.199 (-37.995, 37.596)
Ult. Load	41.549 (-1.545, 84.643)	50.600 (7.344, 93.856)	9.051 (-32.823, 50.925)	10.267 (-33.400, 53.933)	34.092 (-8.910, 77.094)	23.826 (-18.637, 66.289)	1.476 (-42.190, 45.143)	-18.070 (-61.072, 24.932)	-19.546 (-62.009, 22.916)
Toughness	178.177 (-58.880, 415.235)	341.242 (103.627, 578.858)	163.065 (-70.439, 396.569)	67.087 (-171.871, 306.046)	-92.076(-328.870, 144.718)	-159.163 (-394.774, 76.447)	-5.614 (-244.572, 233.345)	-234.127 (-470.921, 2.667)	-228.513 (-464.124, 7.097)
Young's	17.727 (-12.113, 41.567)	21.810 (-5.036, 48.656)	7.083 (-19.722, 33.888)	18.205 (-8.655, 45.065)	5.373 (-21.464, 32.211)	-12.832 (-39.665, 14.000)	-11.580 (-38.440, 15.280)	-6.255 (-33.093,20.582)	5.325 (-21.508, 32.157)
Ult. Tensile Stress	1.463 (-3.197, 6.123)	2.589 (-2.082, 7.261)	1.127 (-3.461, 5.714)	2.780 (-1.919, 7.479)	1.786 (-2.869, 6.441)	-0.994 (-5.624, 3.636)	-1.280 (-5.979, 3.149)	-0.349 (-5.004, 4.306)	0.931 (-3.699, 5.561)
Ult. Strain	-0.111 (-351, 0.129)	-0.035 (-0.276, 0.205)	0.076 (-0.164, 0.316)	-2.337E-5 (-0.240, 0.240)	-0.053 (-0.294, 0.187)	-0.053 (-0.294, 0.187)	0.106 (-0.135, 0.346)	0.027 (-0.213, 0.268)	-0.078 (-0.319, 0.162)

Supplemental Table 9. Comparison of load-to-failure experiments between disease group derived from linear mixed effects model

	VLC YH/DRL YH	VLC YH/POL YH	DRL YH/POL YH	VLC EH/DRL EH	VLC EH/POL EH	DRL EH/POL EH	VLC OA/DRL OA	VLC OA/POL OA	DRL OA/POL OA
	p. adj	*p. adj*	*p. adj*	*p. adj*	*p. adj*	*p. adj*	*p. adj*	*p. adj*	*p. adj*
Stiffness	0.802	**0.043***	**0.002***	0.28	1	0.580	0.526	1	1
Ult. Load	0.87	**0.001***	**<0.001***	**0.006***	0.915	**<0.001***	0.102	0.960	0.668
Toughness	**0.035***	0.086	1	0.587	1	0.604	**0.043***	**<0.001***	0.123
Young's	1	**0.014***	**0.031***	1	1	1	0.664	1	0.351
Ult. Tensile Stress	1	**0.004***	**0.027***	0.429	0.244	1	1	0.332	0.423
Ult. Strain	1	1	0.647	0.144	0.191	1	1	1	1
	Mean diff (95% CI)	*Mean diff (95% CI)*	*Mean diff (95% CI)*	*Mean diff (95% CI)*	*Mean diff (95% CI)*	*Mean diff (95% CI)*	*Mean diff (95% CI)*	*Mean diff (95% CI)*	*Mean diff (95% CI)*
Stiffness	-17.001 (-55.041, 21.040)	39.022 (0.982, 77.063)	56.023 (18.127, 93.919)	-26.059 (-64.107, 11.988)	-6.028 (-44.075, 32.020)	20.032 (-18.203, 58.266)	-20.862 (-58.763, 18.203)	-13.689 (-51.590, 24.212)	7.173 (-30.873, 45.218)
Ult. Load	-14.859 (-49.836, 20.118)	54.376 (19.399, 89.354)	69.235 (33.260, 105.211)	-46.141 (-80.937, -11.346)	14.304 (-20.492, 49.099)	60.445 (26.404, 94.486)	-31.367 (-67.120, 4.386)	-14.294 (-50.047, 21.459)	17.073 (-17.741, 51.887)
Toughness	218.528 (12.026, 425.030)	187.122 (-19.390, 393.624)	-31.406 (-241.996, 179.184)	107.438 (-98.515, 313.390)	3.331 (-202.621, 209.283)	-104.107 (-306.821, 98.608)	-214.791 (-424.711, -4.870)	-388.247 (-598.168, -178.327)	-173.456 (-379.514, 32.601)
Young's	3.416 (-22.846, 29.678)	31.781 (-5.519, 58.044)	28.365 (2.071, 64.660)	6.895 (-19.378, 33.167)	5.474 (-20.798, 31.747)	-1.420 (-27.686, 24.846)	-13.020 (-39.321, 13.280)	3.716 (-22.584, 30.017)	16.737 (-9.534, 43.007)
Ult. Tensile Stress	1.073 (-2.970, 5.116)	5.611 (1.568, 9.655)	4.538 (0.413, 8.664)	2.390 (-1.642, 6.422)	2.869 (-1.163, 6.901)	0.478 (-3.489, 4.446)	0.296 (-3.842, 4.381)	2.679 (-1.439, 6.785)	2.403 (-1.631, 6.438)
Ult. Strain	0.081 (-0.154, 0.316)	-0.038 (-0.273, 0.198)	-0.118 (-0.354, 0.117)	0.191 (-0.044, 0.427)	0.179 (-0.056, 0.414)	-0.013 (-0.247, 0.222)	0.063 (-0.173, 0.298)	0.025 (-0.211, 0.261)	-0.037 (-0.273, 0.198)

Supplemental Table 10. Comparison of load-to-failure experiments between ligament types derived from linear mixed effects model

.

Online Resource 2

Morphological and Mechanical Property Differences in Trapeziometacarpal Ligaments of Healthy and Osteoarthritic Female Joints

Lizzie Walker[1] (mewlkr@g.clemson.edu, ORCiD: 0009-0001-9980-6260), Daniel Gordon[1] (digordo@g.clemson.edu), Alexander Chiaramonti[2] (achiaram@wakehealth.edu), Shangping Wang[1,3] (shagpw@clemson.edu ORCiD: 0000-0003-0049-2212), Zhaoxu Meng[4] (zmeng@clemson.edu ORCiD: 0000-0002-3250-7696), Dane Daley[3] (dalda@musc.edu), Elizabeth Slate[5] (eslate@fsu.edu), Hai Yao[1,3] (haiyao@clemson.edu), Vincent D. Pellegrini, Jr. (vincent.d.pellegrini.jr@dartmouth.edu) [6], Yongren Wu[1,3] (yongren@clemson.edu ORCiD: 0000-0002-5411-8528)

Author Affiliations:

(1) Department of Bioengineering, Clemson University, Clemson, SC
(2) Department of Orthopaedic Surgery and Rehabilitation, Wake Forest University School of Medicine, Winston Salem, NC
(3) Department of Orthopaedics and Physical Medicine, Medical University of South Carolina, Charleston, SC
(4) Department of Mechanical Engineering, Clemson University, Clemson, SC
(5) Department of Statistics, Florida State University, Tallahassee, FL
(6) Department of Orthopaedics, Dartmouth-Hitchcock Medical Center, Lebanon, NH

Corresponding Author: Yongren Wu, yongren@clemson.edu, 843-876-2305, 68 President Street, BEB 203, Charleston, SC, 29425

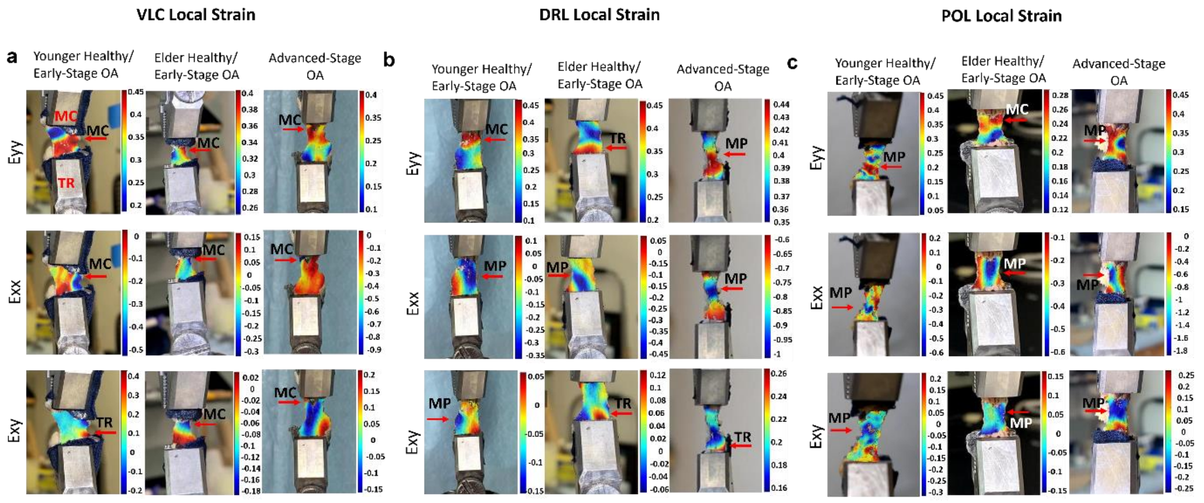

Supplemental Fig. 1 DIC analysis of local strains for (A) VLC, (B) DRL, (C) POL; heat maps for strains in Eyy, Exx, and Exy shown. Arrows denote where failure occurred. MC = metacarpal, MP = midplane, TR = trapezium

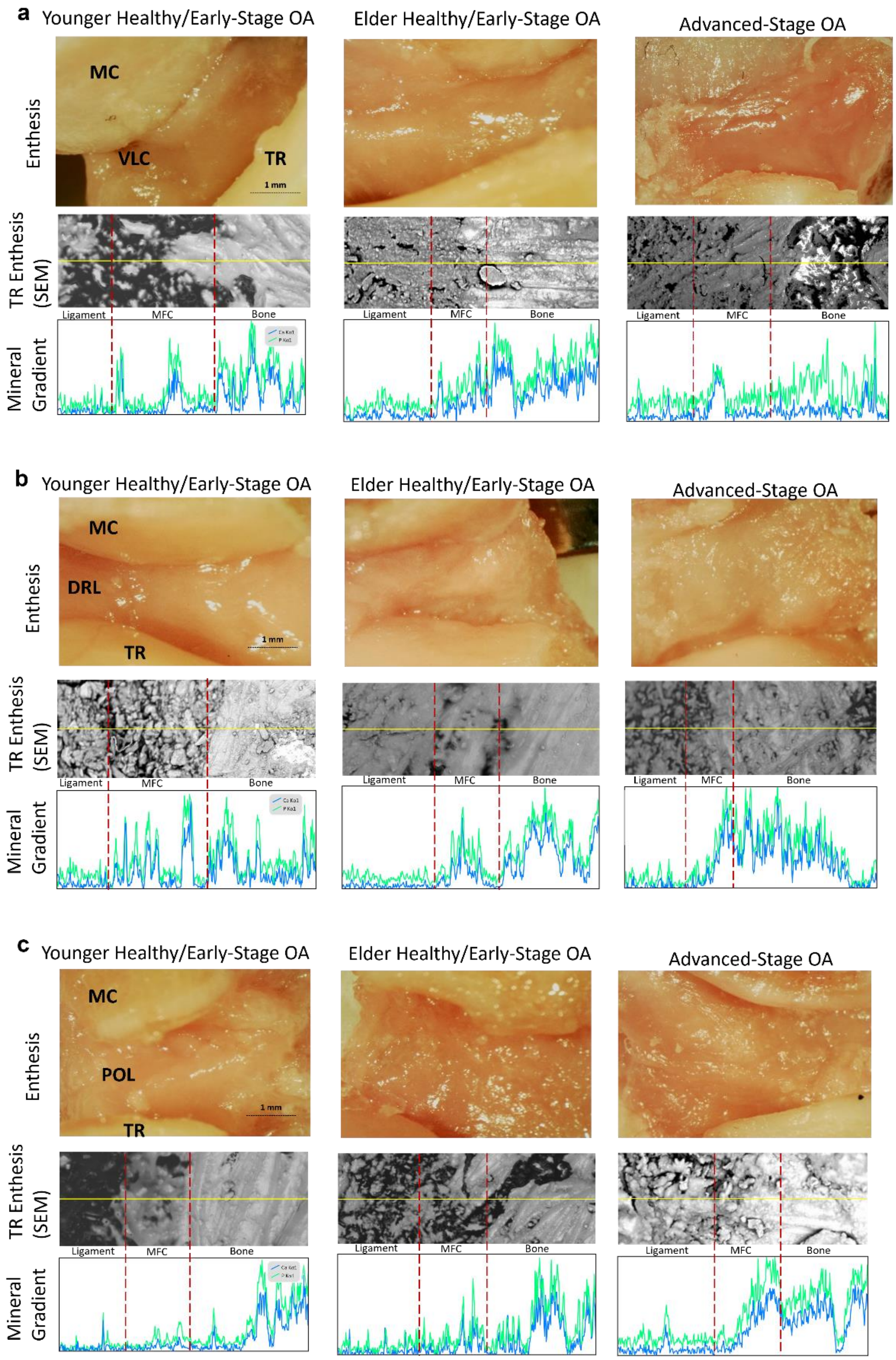

Supplemental Fig. 2 Light microscopy, SEM imaging, and EDS analysis of trapezium insertion of (A) VLC, (B) DRL, (C) POL. Blue represents general levels of calcium, and green represents general levels of phosphorus.
